# Association between helminth infections and diabetes mellitus in adults from the Lao People’s Democratic Republic: a cross-sectional study

**DOI:** 10.1186/s40249-018-0488-2

**Published:** 2018-11-06

**Authors:** Nan Shwe Nwe Htun, Peter Odermatt, Phimpha Paboriboune, Somphou Sayasone, Malisa Vongsakid, Vilayouth Phimolsarn-Nusith, Xuan Duong Tran, Phoum-Savath Ounnavong, Navalone Andriama-Hefasoa, Nilun-Done Senvanpan, Anousine Homsana, Baocher Lianosay, Dalouny Xayavong, Dimbitsoa Rakotomalala Robinson, Phaivanh Bounsavath, Phoy-Phaylinh Prasayasith, Seng-Davanh Syphan, Yi-Xiao Lu, Kanchana Thilakoun, Xaipa-Song Xaiyaphet, Phout-Tasin Vongngakesone, Ikenna C Eze, Medea Imboden, Banchob Sripa, Daniel Reinharz, Nicole Probst-Hensch

**Affiliations:** 1 Department of Epidemiology and Public Health, Swiss Tropical and Public Health Institute, PO Box, 4002, 4051 Basel, Switzerland; 2 University of Basel, Basel, Switzerland; 3 The Francophone Institute for Tropical Medicine (International Program for Health in the Tropics), Vientiane, Lao PDR; 4 Lao Tropical and Public Health Institute, Ministry of Health, Vientiane, Lao People’s Democratic Republic; 5 Tropical Disease Research Laboratory, Faculty of Medicine, Khon Kaen University, Khon Kaen, Thailand; 6 Université Laval, Quebec City, Canada

**Keywords:** Dual burden of disease, *Opisthorchis viverrini*, *Taenia*, Diabetes mellitus, Cross-sectional, Epidemiology, Adults, Lao PDR

## Abstract

**Background:**

As a result of epidemiological transition, the health systems of low- and middle-income countries are increasingly faced with a dual disease burden of infectious diseases and emerging non-communicable diseases. Little is known about the mutual influence of these two disease groups. The aim of this study was to investigate the co-occurrence of helminth infections and diabetes mellitus in adults in Lao People’s Democratic Republic (Lao PDR).

**Methods:**

We conducted a cross-sectional study among 1600 randomly selected adults aged 35 and older from four different socio-economical and ecological provinces. Information on socio-demographics, risk factors and health conditions was obtained from personal interviews. Clinical assessments including anthropometry (height, weight, waist and hip circumference) and blood pressure measurements were also conducted. Diabetes was classified based on self-reported diagnoses and a point-of-care glycated haemoglobin (HbA1c) test from finger prick blood samples. Stool samples for helminth diagnosis were examined with formalin-ether concentration technique for intestinal parasitic infections. The independent associations of helminth infections with diabetic status and HbA1c were assessed using multiple regression analyses.

**Results:**

The prevalence of pre-diabetes and diabetes was 37.3% and 22.8%, respectively. Fifty-six percent of diabetic cases were undiagnosed and 85% of diagnosed diabetic cases had poor glycemic control. Participants from rural areas and from southern parts of the country had higher infection rates, with *Opisthorchis viverrini*, being the most common helminth infection (30.5%). We found a positive association between *Taenia* spp*.* infections and HbA1c (β = 0.117; 95% *CI*: 0.042–0.200) and diabetes mellitus risk (*OR* = 2.98; 95% *CI*: 1.10–8.05). No other helminth species was associated with glycated hemoglobin.

**Conclusions:**

Hyperglycaemia and diabetic rates in Lao PDR are alarmingly high, but consistent with other high rates in the region. Given the high rates of under-diagnosis and poorly-controlled glycaemia in diabetes mellitus patients, routine diabetes screening and treatment is essential for the local healthcare system. Large longitudinal cohorts integrating biomarkers are warranted in the search of causal diabetes mellitus risk factors in the region. Common intestinal helminth infections, including *O. viverrini,* are unlikely to explain the high diabetes mellitus rates observed*.*

**Electronic supplementary material:**

The online version of this article (10.1186/s40249-018-0488-2) contains supplementary material, which is available to authorized users.

## Multilingual abstract

Please see Additional file 1 for translation of the abstract into the five official working languages of the United Nations

## Background

Most low- and middle- income countries (LMICs) are faced with challenges arising from demographic aging and lifestyle changes along with economic development, resulting in an increasing prevalence of non-communicable diseases (NCDs). An estimated 415 million people are currently living with diabetes mellitus (DM) worldwide, and the number is estimated to increase to 642 million in 2040 [1]. Similarly, the infectious disease (ID) burden in these countries also remains high. Approximately, 24% of the world’s population (more than 1.5 billion people) is infected with one or more species of helminth infections [2]. The population of the Lao People’s Democratic Republic (Lao PDR) is affected by even higher rates of helminth infections, as a result of high rates of soil-transmitted helminths (STH) such as hookworm (87%), *Trichuris trichiura* (33%)*, Ascaris lumbricoides* (3%) and *Strongyloides stercoralis* (45%) [3–6]. Foodborne trematode infections are endemic in all provinces in Lao PDR, however rates are particularly high in the southern provinces and frequently reach 50% and above [7]. In a study in the province of Saravane, the prevalence of *Taenia* spp*.* at the village level reached a prevalence of 12% [8]. According to the World Health Organisation (WHO) Lao PDR country profile, 2016, the prevalence of DM in the total population is estimated at 5.6%, and is expected to increase considerably in the near future [9].

Recent epidemiological discoveries have pointed to a potential role of helminth infections in the aetiology of diabetes. Many helminths have evolved to live in human organisms for long periods of time. The strategy against being expelled involves a cross-talk with human innate and adaptive immune responses, which may be mediated in part by alterations in the gut microbiome. The inverse relationship between helminth infections and risk of metabolic disorders has been named “metabolic hygiene hypothesis”, after the framework of the hygiene hypothesis for the association between early childhood infections and allergies. Furthermore, inflammation is an established etiological factor for insulin resistance, a precursor phenotype on the pathway to DM [10]; however, the metabolic hygiene hypothesis has not been commonly tested. The objective of this study was therefore, to evaluate the epidemiological association of specific helminthic infections with glycaemia and DM in adults from the Lao PDR.

## Methods

### Study setting and sample size

A total of 1600 adults aged 35 years and older were recruited in four areas of Lao PDR, each consisting of urban as well as rural sites from the provinces of Vientiane (the Vientiane capital and Pakgneum district), Luang Prabang (LP city and Namback district), Saravane (Saravane city and Saravane district), and Champasack (Pakse city and Kong district), between March and April, 2016. Study subjects were randomly recruited among inhabitants if they had been living in the selected study areas for the last five years. We assumed a 20% parasitic infection rate in the study area. With 186 enrolled individuals the infection rate could be assessed with a 95% confidence interval (*CI*). We compensated for an expected non-optimal compliance by adding 10% to the sample calculated size. Therefore, 200 persons were enrolled in each of the 4 study areas. Data collection took place in March–April 2016. All 1600 participants answered a questionnaire, had a clinical examination and blood taken through finger prick. They also provided two stool samples each. The data collectors were first year master students from the Francophone Institute for Tropical Medicine (IFMT), Vientiane, Lao PDR (today named International Program for Health in the Tropics, now integrated in the Lao Tropical and Public Institute [Lao TPHI] in Vientiane) who had undergone a rigorous training for each segment of data collection.

### Questionnaire and clinical information

Each participant was assigned a specific six-digit identification number, reflecting the study site, the village and the household number. A semi-structured questionnaire was developed to collect information on socio-demographic factors, risk factors for chronic diseases, previous and current health problems, as well as knowledge and perception of diseases of interest for the study. The questionnaire was translated from English to the local language for the interviews.

The clinical examination included a general assessment of the participant’s health status, including the measurement of arterial blood pressure, height, weight, waist and hip circumference, as well as haemoglobin and glycated haemoglobin (HbA1c) in capillary blood.

Blood pressure was measured three times over an interval of five minutes between each reading using the Omron M6 AC (Hoofdoorp, Netherlands) upper arm digital blood pressure monitor. Participants were seated for 15 min before the blood pressure assessment and the mid-arm circumference was also measured with the use of an appropriate semi-rigid preformed arm cuff. The mean systolic and diastolic blood pressure readings were noted.

Height was measured using a SECA 206 roll-up measuring tape with wall attachment. (SECA GmbH & Co. KG, Hamburg, Germany) The height was recorded in centimetres with one decimal.

Weight was measured using the non-automatic personal scale, SECA 877 (SECA GmbH & Co. KG, Hamburg, Germany). Participants were asked to take off shoes, coat and heavy clothes before being weighed. The weight was recorded with one decimal.

Waist and hip circumference was measured using the SECA 203 measuring tape (SECA GmbH & Co. KG, Hamburg, Germany). The waist circumference measured was the area between the ribs and iliac crest, and the hip circumference was the maximum circumference between the iliac crest and the crotch. Measurements were recorded in centimetres with one decimal.

Haemoglobin level was measured using the point-of-care anaemia screening, HemoCue Hb 301 System (HemoCue AB, Ängelholm, Sweden). Results were reported to the nearest 0.1 g/L.

Glycated haemoglobin (HbA1c)/DM status assessment: HbA1c values reflect plasma glucose concentration over an eight to 12 weeks period before the measurement. It is a convenient screening test for diabetes as there is no fasting requirement. HbA1c concentration in blood obtained by a finger-prick test was measured using the point-of-care instrument Afinion AS 100 analyser and cartridges (Alere Inc. Waltham, Massachusetts, USA). The cut-offs of the American Diabetes Association [11] were applied: normal: < 5.7; pre-DM: 5.7–6.4; and DM: ≥6.5. Subjects which self-reported a diagnosis and treatment of DM were classified as DM, irrespective of their HbA1c blood concentration. DM categories were further subdivided into diagnosed and treated DM; diagnosed DM, but untreated DM and undiagnosed DM. Newly diagnosed DM cases with HbA1c levels ≥6.5% were referred to a health clinic or hospital for confirmation of DM.

### Parasitological examination

Two faecal samples were collected consecutively for two days for each participant and fixed in 10% formalin. The fixed samples were sent to the microbiology laboratory at Khon Kaen University, Khon Kaen, Thailand, in which, the formalin ether concentration technique (FECT) was used for helminth diagnosis.

### Data management and analysis

Data was double-entered and subjected to validation checks using EpiData 3.1 (EpiData Association, Odense, Denmark). A complete case analysis (individuals with stool samples, HbA1c results, and complete covariate information) was conducted to investigate the association of explanatory variables (helminths; covariates) with the outcome variable (HbA1c; DM) using STATA 14.1 (StataCorp; College Station, TX, USA). Descriptive statistics of the participants characteristics were reported as frequencies and percentages for categorical variables and means and standard deviations (SD) for continuous variables. The sex-stratified DM categories according to the measured HbA1c concentrations were cross-tabulated with self-reported DM diagnosis and treatment. The helminth infection prevalence was stratified by study sites as well as the type of study area (rural vs urban). The distribution of covariates and helminth infections was presented separately for non-DM (non self-reported DM; normal HbA1c), pre-DM (non self-reported DM; pre-DM HbA1c), and DM groups (self-reported DM diagnosis or diabetic HbA1c). The socio-economic status (SES) of participants was estimated using a household-based asset approach. SES scores were constructed using principle component analysis (PCA). Participants were categorized into three groups; poorest, second poorest and least poorest [12]. Chi-square (*χ*^2^) tests and analysis of variance tests were conducted to investigate the unadjusted association between covariates and DM categories. Linear (outcome HbA1c measurements) and logistic (outcome DM status) regression models with random intercepts of study sites were performed to explore the independent relationship between helminth infections and HbA1c measurements and diabetes, respectively. Participants self-reporting a physician diagnosis of DM and medical DM treatment (*N* = 54) were excluded from these regression analyses. The regression models were adjusted for a set of preselected potential confounders (study sites, age, sex, educational level, SES status, alcohol consumption, smoking status, haemoglobin levels, body mass index [BMI] and physical inactivity). A two-sided *P* value < 0.05 was considered as a statistically significant level.

## Results

**Fig. 1:**
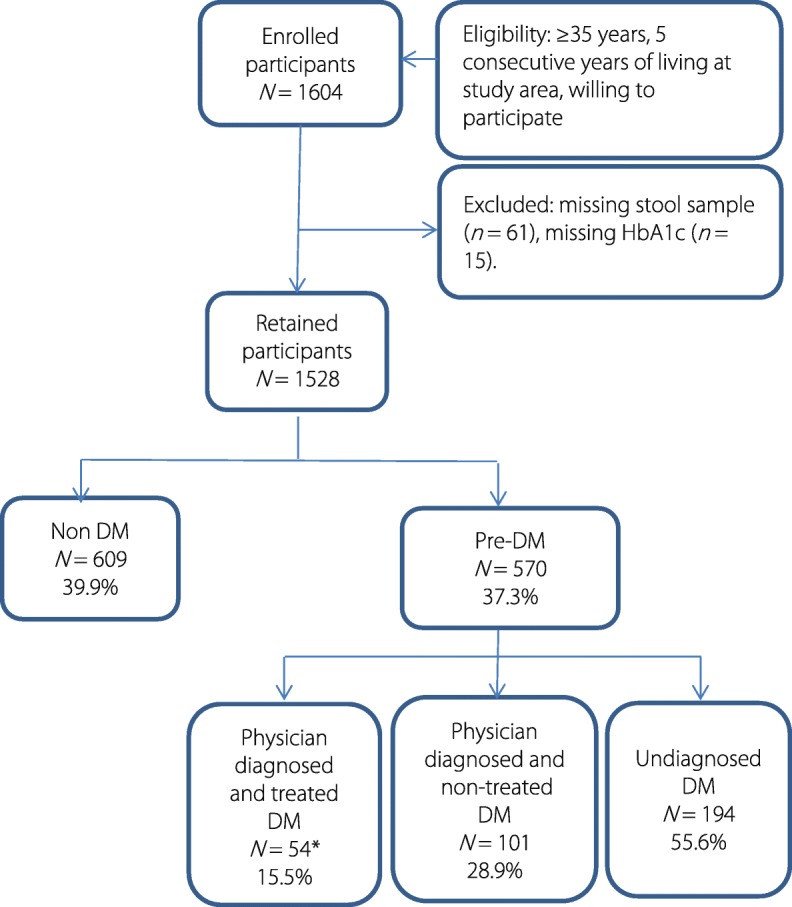
Flow chart of the study sample and distribution of pre-diabetes and diabetes. #categorization based on a positive self-report of DM diagnosis and otherwise based on the HbA1c concentrations. *subjects self-reporting a physician diagnosis and taking DM medication were excluded from multivariable regression models on the association between infections and HbA1c or DM. DM: Diabetes mellitus

Among the 1604 participants, 1528 (95.3%) were considered in the analysis. The observed prevalence of pre-diabetes and diabetes was 37.3% and 22.8%, respectively (Fig. 1). Table 1 summarizes the total and sex-stratified distribution of DM and pre-DM according to HbA1c levels, both, for the entire study sample and stratified by self-reported DM diagnosis and treatment. Among the total sample, 614 participants had HbA1c values in the normal range, in which, five self-reported DM; 585 in the pre-DM range, in which, 15 self-reported DM; and 329 in the DM range, in which, 135 self-reported DM. The distribution of DM categories did not differ considerably between males and females. Fifty-six percent (*n* = 194) participants from a total of 349 participants with DM were unaware of their disease (Fig. 1). Among the 155 participants self-reporting a DM diagnosis, only 39% (*n* = 61) reported some kind of treatment, while only 35% (*n* = 54) reported intake of physician-prescribed anti-diabetic treatment. In more than 85% of patients with self-reported DM, irrespective of undergoing treatment or not, HbA1c concentrations measured were still in the diabetic range.

**Table 1:** Diabetes status according to HbA1c measurements, stratified by sex, self-reported diabetes diagnosis and treatment status

DM category according to HbA1c level^a^	Total *N* (%)	Male *N* (%)	Female *N* (%)
All study participants (*N* = 1528)			
Normal	614 (40.2)	183 (40.9)	431 (39.9)
Pre-DM	585 (38.3)	165 (36.8)	420 (38.9)
DM	329 (21.5)	100 (22.3)	229 (21.2)
Mean (Standard Deviation)	6.4 (1.9)	6.4 (1.9)	6.4 (1.9)
Self-reported DM cases (*N* = 155)			
Normal	5 (3.2)	3 (6.1)	2 (1.9)
Pre-DM	15 (9.7)	3 (6.1)	12 (11.3)
DM	135 (87.1)	43 (87.7)	92 (86.8)
Self-reported DM cases with any treatment (*N* = 61)			
Pre-DM	8 (13.1)	0 (0)	8 (17.0)
DM	53 (86.9)	14 (100)	39 (83.0)
Self-reported DM cases with a physician diagnosis and currently taking DM drugs (*N* = 54)			
Pre-DM	7 (13.2)	0 (0)	7 (17.1)
DM	46 (86.8)	12 (100)	34 (82.9)

^a^According to cut-offs of the American Diabetes Association [11]: Normal: < 5.7; Pre-DM: 5.7–6.4; DM: ≥6.5

*DM,* Diabetes mellitus, *HbA1c* Glycated haemoglobin

Table 2 shows the prevalence of helminth infections stratified by rural and urban areas as well as by the four study provinces. Helminth infections were more frequent in rural areas compared to urban areas and in the two southern provinces compared to the two Northern provinces. The two southern provinces in Lao PDR and along the Mekong River (Saravane and Champasack) had overall infection rates of 49% and 44.9%, respectively, mostly due to *O. viverrini* (43% and 40%, respectively). In fact, *O. viverrini* was the most frequent trematode followed by minute intestinal flukes and *Paragonimus* spp*.* with prevalence rates of 30.5%, 6.3% and 0.3%, respectively. The overall nematodes infection rates were 7.7% including hookworm (4.8%), *S. stercoralis* (2.6%), *T. trichiura* (0.5%) and *A. lumbricoides* (0.3%), respectively. We also found an infection rate of 2.2% for *Taenia* spp.

**Table 2:** Prevalence (*N*, prevalence) of helminth infections in the study area

Helminth infections	Total (*N* = 1528)	Rural (*n* = 766)	Urban (*n* = 762)	Northern Province Vientiane^a^ (*n* = 378)	Northern Province Luang Prabang^a^ (*n* = 363)	Southern Province Saravane^a^ (*n* = 396)	Southern Province Champasack^a^ (*n* = 391)
Any infection	539 (35.3)	332 (43.3)	207 (27.2)	113 (29.9)	58 (16.0)	193 (48.7)	175 (44.8)
Any trematode infection	473 (31.0)	286 (37.3)	187 (24.5)	108 (28.6)	34 (9.4)	174 (43.9)	157 (40.2)
*Opisthorchis viverrini*	466 (30.5)	282 (36.8)	184 (24.1)	106 (28.0)	33 (9.1)	171 (43.2)	156 (39.9)
Minute intestinal flukes	96 (6.3)	71 (9.3)	25 (3.3)	8 (2.1)	0 (0)	45 (11.4)	43 (11.0)
*Paragonimus* spp.	4 (0.3)	2 (0.3)	2 (0.3)	1 (0.3)	1 (0.3)	1 (0.3)	1 (0.3)
Any nematode infection	117 (7.7)	87 (11.4)	30 (3.9)	5 (1.3)	26 (7.2)	52 (13.1)	34 (8.7)
Hookworm	73 (4.8)	55 (7.2)	18 (2.4)	2 (0.5)	10 (2.8)	44 (11.1)	17 (4.4)
*Strongyloides stercoralis*	40 (2.6)	28 (3.7)	12 (1.6)	2 (0.5)	12 (3.3)	9 (2.3)	17 (4.3)
*Ascaris lumbricoides*	4 (0.3)	4 (0.5)	0 (0.0)	1 (0.3)	3 (0.8)	0 (0)	0 (0)
*Trichuris trichiura*	7 (0.5)	5 (0.7)	2 (0.3)	0 (0.0)	4 (1.1)	2 (0.5)	1 (0.3)
Cestodes (*Taenia* spp.)	34 (2.2)	25 (3.3)	9 (1.2)	12 (3.2)	3 (0.8)	13 (3.3)	6 (1.5)

^a^in each province participants from urban and rural sites were recruited

Table 3 compares the characteristic of participants and helminth infection rates across DM status categories, defined by the combination of self-reported DM and HbA1c concentration. The mean age of participants was 54.9 years (SD: 12.0 years, range: 35–95 years), 70% were women, 50% lived in rural settings, 94% belonged to the Lao ethnic group, 16% were illiterate and 31% belonged to the poorest category. With regard to lifestyle factors, 32% reported to be ever smokers, 48% to be ever alcohol drinkers and 19% to be physically inactive. The mean waist circumference was 82.8 cm in men and 80.7 cm in women (recommended cut-off points for Asians: 90 cm in men and 80 cm in women) [13]. The mean Hb concentration was 131.3 mg/dl in men and 119.1 mg/dl in women (WHO cut-off points for non-anaemic Hb values are ≥130 g/L (male); ≥110 g/L (female)). As expected, participants with DM were more likely to be older, to be of higher socio-economic status and to score higher on the anthropometric parameters including BMI, waist circumference, hip circumference and waist-hip ratio. Participants without diabetes exhibited the highest overall infection, with trematode and *O. viverrini* among this unadjusted comparison, whereas there was a tendency for *Taenia* spp*.* to be more common in subjects with DM.

**Table 3:** Participants characteristics according to DM categories (*N* = 1528)

Covariates	Total (*N*, %)	Categories of DM (*N*, %)^a^			*P-value
		No DM (*n* = 609)	Pre-DM (*n* = 570)	DM (*n* = 349)	
Age					< 0.001
35–49 years	535(35.0)	303(49.7)	163(28.6)	69(19.8)	
50–60 years	534(34.9)	177(29.1)	199(34.9)	158(45.3)	
61–95 years	459(30.1)	129(21.2)	208(36.5)	122(34.9)	
Mean, SD	54.9(12.0)				
Female (%)	1080(70.7)	429(70.4)	408(71.6)	243(69.6)	0.83
Rural (%)	766(50.1)	322(52.9)	276(48.4)	168(48.1)	0.12
Ethnicity (Lao)	1438(94.1)	564(92.6)	540(94.7)	334(95.7)	0.59
Education					0.05
Illiterate	247(16.2)	80(13.1)	105(18.4)	62(17.8)	
Primary level	719(47.0)	286(47.0)	270(47.4)	163(46.7)	
Secondary level	562(36.8)	243(39.9)	195(34.2)	124(35.5)	
Socio-economic status					0.002
Poorest	505(33.1)	219(36.0)	199(34.9)	87(24.9)	
Second least poorest	514(33.6)	208(34.1)	186(32.6)	120(34.4)	
Least poorest	509(33.3)	182(29.9)	185(32.5)	142(40.7)	
Ever smokers	491(32.1)	196(32.2)	177(31.1)	118(33.8)	0.64
Ever alcohol drinkers	733(48.0)	354(58.1)	244(42.8)	135(38.7)	< 0.001
No physical activity	288(18.8)	112(18.4)	122(21.4)	54(15.5)	0.13
Weight (kg; mean, SD)	58.1(11.9)	55.6(11.0)	58.3(11.8)	61.9(12.5)	< 0.001
Height (cm; mean, SD)	153.6(7.2)	153.8(7.1)	153.0(7.1)	154.0(7.4)	0.08
BMI (kg/m^2^; mean, SD)	24.6(4.5)	23.5(4.1)	24.8(4.5)	26.0(4.5)	< 0.001
Hip circumference (cm; mean, SD)	93.2(10.0)	91.0(9.7)	94.1(10.0)	95.8(9.7)	< 0.001
Waist circumference(cm; mean, SD)	81.2(11.8)	77.4(10.8)	82.3(11.7)	86.5(11.2)	0.12
Waist-hip ratio (mean, SD)	0.87(0.07)	0.85(0.06)	0.87(0.06)	0.90(0.06)	< 0.001
Hb concentration (mg/dl; mean, SD)	122.7(17.8)	122(18)	123(18)	125(18)	0.04
Helminth infections					
Any infection	513(33.6)	224(36.8)	189(33.2)	100(28.7)	0.04
Any trematode infection	473(31.0)	211(34.6)	175(30.7)	87(24.9)	0.03
Opisthorchis viverrini	466(30.5)	207(34.0)	169(29.6)	90(25.8)	0.03
Minute intestinal flukes	96(6.3)	37(6.1)	41(7.2)	18(5.2)	0.62
*Paragonimus* spp*.*	4(0.3)	1(0.2)	2(0.3)	1(0.3)	0.82
Any nematode infection	117(7.7)	51(8.4)	49(8.4)	17(4.9)	0.16
Hookworm	73(4.8)	34(5.6)	25(4.4)	14(4.0)	0.23
*Strongyloides stercoralis*	40(2.6)	13(2.1)	21(3.7)	6(1.7)	0.15
*Ascaris lumbricoides*	4(0.3)	2(0.3)	2(0.3)	0(0.0)	0.56
*Trichuris trichiura*	7(0.5)	4(0.7)	3(0.5)	0(0.0)	0.34
Cestodes/*Taenia* spp.	34(2.2)	10(1.6)	11(1.9)	13(3.7)	0.08

* *P*-value comparing the distribution of the respective factor between DM categories

^a^categorization based on a positive self-report of DM diagnosis and otherwise based on the HbA1c concentrations

*DM* Diabetes mellitus

Table 4 shows the independent associations of infection groups and single infections with HbA1c in the study population after exclusion of subjects with physician-diagnosed and treated DM. Results are presented with and without adjustment for BMI and physical inactivity, which may in part be mediators of the association between helminth infections and HbA1c. Positive associations between *Taenia* spp. infections and HbA1c were observed, in both, models containing infection groups of single infections and models with and without adjustment for BMI and physical activity (largest effect estimate in model of infections groups and adjusting for BMI and physical activity: β = 0.117; 95% *CI*: 0.042–0.200). Interestingly, this positive association seems to be driven by an association with HbA1c in the diabetic range. In healthy subjects without pre-DM or DM, we found an inverse relationship between *Taenia* spp. and HbA1c measurements (β = − 0.049; 95% *CI*: -0.075–0.022) (Additional file 2). No association of HbA1c with any other infection or infection group was observed.

**Table 4:** Independent association of single infections and infection groups with HbA1c in all participants^a^

Infections	Adjusted for other infections, study site, age, sex, SES status, education status, smoking status, alcohol consumption and haemoglobin level		Additionally adjusted for BMI and physical inactivity	
	β	95% *CI*	β	95% *CI*
Grouped infections^b^ (*N* = 1474)				
Nematodes	−0.040	−0.081–0.002	−0.035	−0.076–0.006
Trematodes	−0.003	−0.027–0.021	−0.0003	−0.023–0.024
Cestodes *Taenia* spp.	0.112	0.037–0.188	0.117	0.042–0.200
Single infection^c^ (*N* = 1466)^d^				
Hookworm	−0.030	−0.083–0.021	−0.025	−0.076–0.027
*Opisthorchis viverrini*	−0.009	− 0.035–0.017	− 0.002	− 0.025–0.022
Minute intestinal flukes	0.021	− 0.028–0.069	0.011	− 0.034–0.056
*Strongyloides stercoralis*	−0.043	− 0.128–0.014	− 0.046	−0.116–0.024
*Trichuris trichiura*	−0.043	− 0.202–0.115	−0.063	− 0.220–0.094
*Taenia* spp*.*	0.114	0.039–0.190	0.116	0.042–0.192

^a^Participants excluding participants self-reporting a physician-diagnosis of DM and intake of DM medication

^b^Model including infection groups nematodes (yes vs no), trematode infection (yes vs. no), and cestodes (yes vs no)

^c^Model including single infections hookworm (yes vs no), *O. viverrini* (yes vs no), minute intestinal flukes (yes vs no), *Strongyloides stercoralis* (yes vs no), *Trichuris trichiura* (yes vs no) and *Taenia* spp*./*cestodes (yes vs no)

^d^Subjects with other rare types of infections were excluded from this analysis

*CI*: Confidence interval

Table 5 shows the independent associations of single helminth infections with DM compared to non-DM after excluding subjects with a self-reported physician diagnosis of DM and intake of DM medication, and leaving the pre-DM category out. Results are again presented with and without adjustment for BMI and physical inactivity. Consistent with our findings on the infection-HbA1c associations, we found that having a Cestode infection, but not any other infections was associated with an increased risk of DM (Adjusted *OR* = 2.98, 95% *CI*: 1.10–8.05). The independent association of other factors with DM risk in the expected directions validate the assessment of DM and HbA1c: age (*OR* = 1.05, 95% *CI*: 1.04–1.08), being male (*OR* = 1.42, 95% *CI*: 0.89–2.00), highest socio-economic status (*OR* = 1.70, 95% *CI*: 1.08–2.67), ever smoker (*OR* = 1.89, 95% *CI*: 1.22–2.92), and exhibiting high Hb concentration (*OR* = 1.02, 95% *CI*: 1.15–1.25), and high BMI (*OR* = 1.20, 95% *CI*: 1.15–1.25). Interestingly, self-reported alcohol consumption (*OR* = 0.54, 95% *CI*: 0.38–0.76) was inversely associated with DM risk. These results did not substantially change when pre-DM and DM were jointly compared to the pre-DM or DM group (Additional file 3).

**Table 5:** Independent association of single infections with DM compared to non-DM, with and without adjustment for BMI and physical inactivity in all participants (*N* = 892^a^)

DM status compared to Normal^b^	*OR* ^c^	95% *CI*	*P*-value	*OR* ^d^	95% *CI*	*P*-value
Hookworm	0.79	0.36–1.72	0.56	0.94	0.42-2.10	0.88
*Opisthorchis viverrini*	0.76	0.52–1.10	0.15	0.87	0.58–1.28	0.46
Minute intestinal flukes	1.38	0.68–2.78	0.37	1.20	0.57–2.52	0.63
*Strongyloides stercoralis*	0.65	0.19–2.30	0.51	0.65	0.15–2.72	0.55
*Trichuris trichiura*	1.00	Omitted		1.00	Omitted	
*Taenia* spp.	2.59	0.98–6.87	0.06	2.98	1.10–8.05	0.03
Study sites(Vientiane as reference)						
Lung Prabang	0.75	0.47–1.20	0.24	0.80	0.48–1.31	0.37
Saravane	0.74	0.47–1.28	0.32	0.89	0.52–1.51	0.66
Champasack	0.76	0.48–1.20	0.24	0.85	0.52–1.39	0.53
Socio-economic status(Poorest as reference)						
Second least poorest	1.51	1.00–2.27	0.04	1.30	0.84–1.99	0.24
Least poorest	2.16	1.42–3.31	< 0.001	1.70	1.08–2.67	0.02
Age	1.05	1.04–1.07	< 0.001	1.05	1.04–1.08	< 0.001
Gender (Male)	1.64	1.05–2.56	0.03	1.42	0.89–2.27	0.14
Education(Illiterate as reference)						
Primary	0.92	0.57–1.47	0.72	0.71	0.43–1.18	0.19
Secondary	0.85	0.49–1.45	0.55	0.69	0.39–1.21	0.20
Ever smokers	1.53	1.01–2.32	0.04	1.89	1.22–2.92	0.004
Ever alcohol drinkers	0.54	0.38–0.76	< 0.001	0.48	0.33–1.03	< 0.001
Hb concentration	1.02	1.01–1.03	< 0.001	1.02	1.01–1.03	0.002
+ BMI	–	–	–	1.20	1.15–1.25	< 0.001
+ Physical activity	–	–	–	1.19	0.76–1.87	0.45

^a^Subjects with pre-DM and participants self-reporting a physician diagnosis of DM and intake of DM medication and with other rare types of infections were excluded from this analysis

^b^Categorization based on a positive self-report of DM diagnosis and otherwise based on the HbA1c concentrations

^c^Mutually adjusted for variables listed

^d^Additionally adjusted for BMI and physical inactivity

*CI* Confidence interval, *OR* Odd ratio, *BMI* Body mass index, *DM* Diabetes mellitus

## Discussion

We found alarmingly high rates of DM and pre-DM in this adult study population in Lao PDR. Local population-based HbA1c findings for comparison are not available in Lao PDR, however, the prevalence found in this study is consistent and comparable to that of the province of Guangdong, an economically well-developed and urbanized area in Southern China, in which the prevalence is 22% [14]. The study in China estimated the diabetes prevalence based on a combination of self-reported DM, HbA1c measured in capillary finger-prick blood as well as venous blood fasting glucose and glucose tolerance testing. This suggests that the observed DM prevalence in Lao PDR, may even be higher than reported here as adding glucose to the DM screening tests results in the identification of additional cases [14, 15]. The high pre-DM and DM rates with a tendency for earlier onset in Asian populations remain partly unexplained [16]. According to the results of this current study common helminth infections in Lao PDR may not explain the high DM and pre-DM prevalences.

The current findings on the prevalence and distribution of helminth infections are consistent with previous studies in Lao PDR. Multiple helminth infections of different trematode, nematodes and cestode species were also common as documented in previous literature [17–20]. The high prevalence rates of the trematode infection, particularly with *O. viverrini,* is consistent with previous studies [21]. Minute intestinal flukes and hookworm were also very frequently diagnosed. These helminths are generally more common in Lao PDR and the Mekong sub-region [19, 22]. In our study we found 2.2% participants infected with *Taenia* spp*.,* which is in general a rather high rate but comparable to previous observations from our team [23]. The national deworming program from the Ministry of Health implements biannual treatment of soil-transmitted helminths in school-children in collaboration with the ministry of education. In recent years, several rounds of mass-treatment were conducted with praziquantel in selected districts of Southern Lao PDR. While these control measures have had a considerable impact on the infection rates of soil-transmitted helminths, the prevalence of liver fluke infections were not reduced.

We have previously reported highly prevalent liver pathologies in rural Lao PDR, as documented by an ultrasound-based study in Saravane province, one of the current study areas [24]. We associated severe liver morbidity with *O. viverrini* [21], known to be a main risk factor for cholangiocarcinoma [25, 26]. The absence of an association between *O. viverrini* infection and DM risk, suggests that *O. viverrini* related liver pathology may not contribute to DM development and the high rate of hyperglycaemia. In contrast, fatty liver disease, especially non-alcoholic fatty liver disease, is viewed as hepatic manifestation of the metabolic syndrome, is associated with insulin resistance, and was previously found to be an independent predictor of incident type 2 DM [27], due to chronic inflammation of the liver or hepatokine secretion. Fatty liver disease and *O. viverrini* associated liver pathology are different entities, which can in principle be differentiated by ultrasound, but the histological examination of liver biopsies remains the diagnostic gold standard. The independent and combined effects of *O. viverrini* infections, associated liver pathologies and fatty liver disease on incident DM need further investigation as the interactions are likely complex and possible in opposite directions. Future studies should also consider *O. viverrini* related modifications of the gut microbiome given the strong evidence for its relation to type 2 DM [28, 29].

Both HbA1c and DM status were positively associated with *Taenia* spp. infection. Taeniasis is an intestinal parasitic infection and is acquired by the consumption of raw or undercooked meat (pork or beef) [30]. It is common in developing countries of Latin America, Asia, and Africa, and associated with poor hygienic and sanitary conditions (inadequate use of latrines or open-air defecation, traditional pig farming, lack of regulation on meat inspection and inadequate water supply) [30]. *Taenia* spp. infection is listed as one of the 17 neglected zoonotic diseases by WHO, which can be preventable and treatable. Individuals with intestinal *Taenia* infection are usually asymptomatic. *T. solium* is the cause of cysticercosis if parasite eggs are faecal-orally acquired. Cysticercosis may lead to a severe disease, particularly if the central neural system (neuro-cysticercosis) is affected [31]. There was no diagnosis of cysticercosisin our study sample.. All taeniasis patients in our study had a history of raw meat consumption (92% beef and 32% pork). People infected came mostly from rural areas of Vientiane and Saravane provinces. Co-infections with other helminths were also detected in half of *Taenia* sp. infected individuals, mostly with *O. viverrini* and/or minute intestinal flukes, hookworm and *S. stercoralis*. One study examining the effect of immune modulation induced by *Taenia crassiceps* infection on the outcome of multiple low dose of Streptozotocin-induced diabetes (MLDS) reported that *T. crassiceps* infection might protect against MLDS, irrespective of the host’s genetic background. To the best of our knowledge, this is the first study to report an association between *Taenia* sp. infection and DM as well as HbA1c.

A limited number of studies have started to explore the interrelation between helminth infections and diabetes. Endemic helminth infections are thought and in part shown to affect insulin sensitivity and resistance through immune-modulating properties and by reducing energy intake and altering energy balance [32]. Yet, epidemiological evidence remains poor, and inconsistent, and points to infection-specific associations with DM. Some previous studies have reported a negative association between soil-transmitted helminth infections and insulin resistance [33], between filarial infection and type 1 and type 2 DM [34], and between *Schistosoma japonicum* infections and type 2 DM [35]. An infection with *S. stercoralis* was found positively associated with severe DM [36]. We reported a positive association of *H. pylori* infections with HbA1c in school children from poor neighbourhoods in South Africa, but neither a cross-sectional association with other common helminth infections nor a change in HbA1c as a result of anti-helminthic treatment [37]. It has been shown that socioeconomic, environmental and behavioural factors influence the prevalence and intensity of helminth infections and could therefore, in part, be confounders as well as mediators of any association with diabetes [18, 38]. It is thus, important to consider the recently published results from the first randomized placebo-controlled SUGARSPIN trial, investigating the effect of anthelmintic treatment on whole-body insulin sensitivity in a large Indonesian population sample [39]. Albendazole treatment had no effect among participants without any or only a single species helminth infection measured (hookworm [*Ancylostoma duodenale*, *Necator americanus*]; *A. lumbricoides; T. trichiura*; *Strongyloides stercoralis*), but resulted in high homeostatic model of insulin resistance, a measure of insulin resistance, in the presence of multiple species infection at baseline. No effect on HbA1c was observed, though.

Our study has a number of strengths. First, this is the only study to date assessing DM prevalence in Lao PDR, based on Hba1c measurements. Participants from the rural and urban area of the provinces located in the middle and southern part of the country were included; therefore, the findings reflect the prevalence of helminths and DM status of different populations from different geographical settings. Access to curative health services is very low in rural settings. Therefore, our results underline the needs for peripheral curative health services for DM diagnosis and management. Second, no previous study investigated the dual burden of diseases of DM and helminth infections in adults in Lao PDR. In fact, this is the first time the association of *O. viverrini* infection with DM was investigated. Third, we used an internationally certified HbA1c test, which was able to capture most of the previously diagnosed DM cases. The HbA1c point-of-care method applied was validated for use in hot and remote low-income settings in the context of our previous study in South Africa [37]. The validity of our HbA1c findings is further supported by their positive association with validated DM risk factors such as age, BMI and smoking. Finally, to achieve a satisfactory sensitivity for the helminth diagnosis, we examined two stool samples per person, which is known to increase the sensitivity [40].

There are however, some limitations in our study. It is known that *T. solium*, *T. saginata* and *T. asiatica* are endemic in Asia [41]. In our study, we did not distinguish the *Taenia* species, and hence could not study the species specific associations with HbA1c in the blood. Moreover, due to the cross-sectional nature of our study, we were not able to identify the time-course relationship between infections and DM development. Reverse causation is a concern as DM patients have an increased susceptibility to infections due to their immune dysfunction [42]. The observed association between *Taenia* spp*.* and HbA1c could therefore be real, a chance finding, explained by confounding or due to reverse causation. Finally, we could not rule out other associated infectious or non-infectious conditions of study participants, which might influence the blood glucose levels and confound, modify or mediate the observed infection-DM associations. Multi-parasitism is very common in different provinces in Lao PDR and a significant association has been observed between *S. mekongi* and hookworm in Southern Lao PDR [23], as well as between *S. mansoni* and hookworm in Côte d’Ivoire [43]. Since each parasite has a different effect on blood sugar, energy balance, and immunity, co-infections status may play an important role in studying dual disease burden.

## Conclusions

Our study found an alarmingly high level of uncontrolled hyperglycaemia in both, urban and rural Lao PDR. These results are not consistent, with a strong role of common infections, such as *O. viverrini,* explaining the high diabetes burden observed. Larger and longitudinal studies including biomarkers and liver ultrasounds are warranted to further study the causes of DM in Lao PDR. The Lao PDR health system must work on strengthening its healthcare services in the domain of DM screening and treatment.

## Additional files

Additional file 1: Multilingual abstract in the five official working languages of the United Nations. (PDF 272 kb)

Additional file 2: Independent association of single infections and infection groups with HbA1c in participants with HbA1c levels in the normal range (columns to the left) (*N* = 609) and in the normal and prediabetic range (columns to the right) (*N* = 1179), excluding participants self-reporting a physician-diagnosis of DM. (PDF 80 kb)

Additional file 3: Independent association of single infections with pre-DM and DM status combined and compared to non-DM, with and without adjustment for BMI and physical inactivity, excluding participants self-reporting a physician diagnosis of DM and intake of DM medication (*N* = 1466^1^) (PDF 80 kb)

## Abbreviations

BMI: Body mass index
*CI*: Confidence interval
DM: Diabetes mellitus
FECT: Formalin-ether concentration technique
ID: Infectious diseases
IFMT: The Francophone Institute for Tropical Medicine
Lao PDR: Lao People’s Democratic Republic
LMICs: Low- and middle income countries
NCDs: Non-communicable diseases
*OR*: Odd ratio
SD: Standard deviation
STH: Soil-transmitted helminths
WHO: World Health Organization
