# Supplementary material for: Association between helminth infections and diabetes mellitus in adults from the Lao People’s Democratic Republic: a cross-sectional study
Source: Infect Dis Poverty. 2018 Nov 6;7:105. doi: 10.1186/s40249-018-0488-2 (PMC6219195; doi:10.1186/s40249-018-0488-2)
Supplement: Supplementary file 1 — Multilingual abstract in the five official working languages of the United Nations. (PDF 272 kb) [file 40249_2018_488_MOESM1_ESM.pdf]

Translation of the abstract into the five official working languages of the United Nations

لاقة عدوى الديدان الطفيلية ومرض السكرى لدى البالغين فى جمهورية لاوس الديمقراطية الشعبية : دراسة شاملة لعدة قطاعات

Nan Shwe Nwe Htun, Peter Odermatt, Phimpha Paboriboune, Somphou Sayasone, Melisa Vongsakid, Phimolsarn-Nusith Vilayouth, Xuan Duong Tran, Phoum-Savath Younnavong, Navalone Andriama-Hefasoa, Nilun-Done Senvanpan, Anousine Homsana, Baocher Lianosay, Dalouny Xayavong, Dimbitsoa Rakotomalala Robinson, Phaivanh Bounsavath, Phoy-Phaylinh Prasayasith, Seng-Davanh Syphan, Yi-Xiao Lu, Kanchana Thilakoun, Xaipa-Song Xaiyaphet, Phout-Tasin Yongngakesone, Ikenna C. Eze, Medea Imboden, Banchob Sripa, Daniel Reinharz and Nicole Probst-Hensch

مُلَخَّصٌ

المعلومات الأساسية:نتيجة لعملية التحول الوبائي تواجه الأنظمة الصحية في الدول ذات الدخل المحدود والمتوسط بشكل متزايد عبئاً مزدوجاً من الأمراض المعدية والأمراض الناشئة غير المعدية. فلا توجد معلومات كافية حول التأثير المشترك لهاتين المجموعتين. وهدفت هذه الدراسة للتحقق من مدى إصابة البالغين بهذين المرضين بشكل متزامن في جمهورية لاو الديمقراطية الشعبية (جمهورية لاو).

المنهجية: قمنا بإجراء دراسة مستعرضة على 1600 شخصًا بالغًا ممن تبلغ أعمارهم 35 سنة فما فوق، وقد تم اختيارهم بطريقة عشوائية من أربعة أقاليم مختلفة اجتماعيًا واقتصاديًا وبيئيًا. وقد تم الحصول على المعلومات المتعلقة بالحالات الاجتماعية الديموغرافية وعوامل الخطورة والحالات الصحية عن طريق المقابلات الشخصية. كما تم إجراء الفحوصات السريرية التي تتضمن القياسات الانثروبمترية (كالطول والوزن ومحيط الخصر والأرداف) وكذلك قياسات ضغط الدم. وقد صُنّف مرض السكري بناءً على التشخيصات التي أبلغ عنها الأفراد وعلى نتائج فحص الهيموجلوبين الذي أجري في نقطة الرعاية على عينات الدم المأخوذة من الإصبع. كما تم فحص عينات البراز الخاص بتشخيص الإصابة بالديدان الطفيلية بواسطة تقنية تركيز الفورمالين الإثير للعدوى الطفيلية المعوية. وقد تم تقييم العلاقات المستقلة الخاصة بإصابات العدوى الطفيلية مع حالات مرض السكري وفحص الهيموجلوبين السكري بواسطة استخدام تحاليل الانحدار المتعدد.

نتائج الدراسة: بلغت الحالات المعرّضة للإصابة بالسُكري 37.7%، بينما بلغت الحالات المُصابة به فعليًا نحو 22.8%. ولم يتم تشخيص 56% من حالات الإصابة بالسكري، وقد أظهرت 85% من الحالات المُشخَّصة بالسكري ضعفًا في ضبط مستوى السكر في الدم. وقد أظهرت فئة الدراسة ممن يسكنون المناطق الريفية والأجزاء الجنوبية من البلاد معدلات مرتفعة في العدوى، وكان متأخر الخصية الزبادي الأكثر شيوعا من بين حالات عدوى الديدان الطفيلية بنسبة (30.5%).

دقو تبث دوجو ةقلاع ةيباجيإ نيب </g>ىودع نادیدلا ةيطيرشلا</g> ءلازعلا <g id="6">والهيموجلوبين السكري</g> (β = 0.117; 95% </g>CI<g id="7"> 0.042–0.200) رطخو ةباصلإا ضرمب يركسلا (OR<g id="8"> = 2.98; 95% <g id="9">CI</g>: 1.10–8.05</g>).الأخرى والهيموجلوبين السكري.

الاستنتاجات: وجد أن معدلات فرط السكر في الدم وحالات الإصابة بمرض السكري مرتفعة على نحو مثير للقلق في جمهورية لاو، إلا أن هذه المعدلات تتوافق مع المعدلات المرتفعة الأخرى في المنطقة. ففي ظل المعدلات المرتفعة لضعف تشخيص السكر في الدم وصعوبة ضبطه لدى مرضى السكري فإن فحوصات مرض السكري الروتينية ومعالجته تعد ضرورة ملحة لنظام الرعاية الصحية المحلي. ويستدعي هذا الأمر دراسة مجموعات طولية كبيرة تتضمن المؤشرات الحيوية عند البحث في عوامل الخطر المؤدية إلى الإصابة بمرض السكري في المنطقة. ومن غير المحتمل أن تفسر عدوى الديدان الطفيلية المعوية الشائعة -ومن ضمنها متأخر الخصية الزبادي- سبب المعدلات المرتفعة لحالات مرض السكري التي تم رصدها.

Translated from English version into Arabic by Khaled Al-Shehari and Mohamed Fouad, through

TRANSLATORS
WITHOUT BORDERS

老挝成人中蠕虫感染与糖尿病的相关性：一项横断面研究

Nan Shwe Nwe Htun, Peter Odermatt, Phimpha Paboriboune, Somphou Sayasone, Melisa Vongsakid, Phimolsarn-Nusith Vilayouth, Xuan Duong Tran, Phoum-Savath Younnavong, Navalone Andriama-Hefasoa, Nilun-Done Senvanpan, Anousine Homsana, Baocher Lianosay, Dalouny Xayavong, Dimbitsoa Rakotomalala Robinson, Phaivanh Bounsavath, Phoy-Phaylinh Prasayasith, Seng-Davanh Syphan, Yi-Xiao Lu, Kanchana Thilakoun, Xaipa-Song Xaiyaphet, Phout-Tasin Yongngakesone, Ikenna C. Eze, Medea Imboden, Banchob Sripa, Daniel Reinharz and Nicole Probst-Hensch

摘要

引言：随着流行病学转变，中低收入国家的卫生系统正逐渐承受着更多的传染性疾病和新发非传染性疾病带来的双重疾病负担。但目前关于这两类疾病相互影响的研究较少。本研究旨在调查老挝人民民主共和国（Lao PDR）成人中蠕虫感染和糖尿病的并发情况。

方法：我们选取了四个具有社会经济和生态差异的省份，并从中随机纳入的 1600 名 35 岁及以上

的成年人进行了横断面研究。本研究通过个人访谈获得社会人口统计学、风险因素和健康状况的相关信息；对人体测量数据（身高、体重、腰围和臀围）和血压数值进行了临床评估；根据自述诊断和指血样本的 HbA1c 测试对糖尿病进行分类；采用福尔马林–乙醚浓集方法对粪便样本进行肠道寄生虫检测。本研究采用多元回归分析法评估了蠕虫感染与糖尿病状态和 HbA1c 之间的相关性。
结果：糖尿病前期和糖尿病患病率分别为 37.3％和 22.8％。56％的糖尿病病例未被诊断，85％的确诊糖尿病患者血糖控制不佳。来自该国农村和南部地区的受试者中的蠕虫感染率较高，其中感染最多的是麝猫后睾吸虫（30.5％）。 我们发现感染绦虫属寄生虫同 HbA1c（β= 0.117；95％ *CI*：0.042-0.200）和罹患 DM 风险（*OR* = 2.98；95％ *CI*：1.10-8.05）之间存在正相关。其它蠕虫感染与 HbA1c 没有相关性。
结论：老挝高血糖症和糖尿病的发病率非常高，与该地区其它疾病的高发病率一致。鉴于 DM 患者的诊断率低和血糖控制较差，当地医疗卫生系统有必要进行常规 DM 筛查和治疗。在研究该地区的 DM 因果风险因素时有必要进行整合生物标志物的大型纵向队列研究。常见的肠道蠕虫感染，包括麝猫后睾吸虫，不太可能用于解释观察到的高 DM 发病率。

Translated from English version into Chinese by Peng Song, edited by Jin Chen

CDC CHINESE CENTER FOR DISEASE CONTROL AND PREVENTION
NATIONAL INSTITUTE OF PARASITIC DISEASES

Association entre les helminthiases et le diabète sucré chez les adultes de la République Démocratique Populaire Lao : une étude transversale

Nan Shwe Nwe Htun, Peter Odermatt, Phimpha Paboriboune, Somphou Sayasone, Melisa Vongsakid, Phimolsarn-Nusith Vilayouth, Xuan Duong Tran, Phoum-Savath Younnavong, Navalone Andriama-Hefasoa, Nilun-Done Senvanpan, Anousine Homsana, Baocher Lianosay, Dalouny Xayavong, Dimbitsoa Rakotomalala Robinson, Phaivanh Bounsavath, Phoy-Phaylinh Prasayasith, Seng-Davanh Syphan, Yi-Xiao Lu, Kanchana Thilakoun, Xaipa-Song Xaiyaphet, Phout-Tasin Yongngakesone, Ikenna C. Eze, Medea Imboden, Banchob Sripa, Daniel Reinharz et Nicole Probst-Hensch

Résumé
Contexte : Du fait de la transition épidémiologique, les systèmes de santé des pays à revenu faible et intermédiaire sont de plus en plus confrontés à une double charge de morbidité due aux maladies infectieuses et aux maladies non transmissibles émergentes. On ne connait que peu de choses sur l'influence mutuelle de ces deux groupes de maladies. Le but de cette étude était d'analyser la cooccurrence d'infections à helminthes et de diabète sucré chez des adultes en République Démocratique Populaire Lao (RDP Lao).
Méthodes : Nous avons mené une étude transversale auprès de 1600 adultes âgés de 35 ans et plus, sélectionnés de façon aléatoire et provenant de quatre provinces socio-économiques et écologiques différentes. Des informations sur les caractéristiques socio-démographiques, les facteurs de risque et les conditions de santé ont été obtenues à partir d'entretiens personnels. Des évaluations cliniques comprenant l'anthropométrie (taille, poids, tour de taille et tour de hanche) et des mesures de la pression artérielle ont également été réalisées. Les diabètes ont été classés sur la base des diagnostics auto-déclarés et d'un test HbA1c effectué sur le lieu de soins à partir d'échantillons de sang prélevés par piqûre au doigt. Des échantillons de selles pour le diagnostic des helminthes ont été examinés avec une technique de concentration formol-éther pour les infections parasitaires intestinales. Les associations indépendantes d’helminthiases avec statut diabétique et HbA1c ont été évaluées à l'aide d'analyses de régression multiples.
Résultats: La prévalence du pré-diabète et du diabète était respectivement de 37,3% et 22,8%. Cinquante-six pour cent des cas de diabète étaient non diagnostiqués et 85% des cas de diabète diagnostiqués avaient un mauvais contrôle glycémique. Les participants des zones rurales et méridionales du pays avaient des taux d’infection plus élevés, avec l’*Opisthorchis viverrini*, étant l’infection à helminthes la plus fréquente (30,5%). Nous avons trouvé une association positive entre les infections *Taenia* spp. et HbA1c (β = 0,117; 95% *CI*: 0,042–0,200) et risque DM (*OR* = 2,98; 95% *CI*: 1.10–8.05). Aucune autre espèce d'helminthes n'était associée à l'HbA1c.
Conclusions: Les taux d'hyperglycémie et de diabète en RDP Lao sont alarmants, mais cohérents aux autres taux élevés dans la région. Compte tenu des taux élevés de sous-diagnostic et de glycémie mal

contrôlée chez les patients DM, un dépistage et un traitement DM systématiques sont essentiels pour le système de santé local.. Les grandes cohortes longitudinales intégrant des biomarqueurs sont garanties dans la recherche de facteurs de risque de DM dans la région. Les helminthiases intestinales courantes, y compris l'*O. viverrini*, sont peu susceptibles d'expliquer les taux élevés de DM observés.

Translated from English version into French by Edith Taki and Laurine CREVOISIER, through

Связь между гельминтозами и сахарным диабетом у взрослых из Лаосской Народно-Демократической Республики: кросс-секционное исследование

Nan Shwe Nwe Htun, Peter Odermatt, Phimpha Paboriboune, Somphou Sayasone, Melisa Vongsakid, Phimolsarn-Nusith Vilayouth, Xuan Duong Tran, Phoum-Savath Younnavong, Navalone Andriama-Hefasoa, Nilun-Done Senvanpan, Anousine Homsana, Baocher Lianosay, Dalouny Xayavong, Dimbitsoa Rakotomalala Robinson, Phaivanh Bounsavath, Phoy-Phaylinh Prasayasith, Seng-Davanh Syphan, Yi-Xiao Lu, Kanchana Thilakoun, Xaipa-Song Xaiyaphet, Phout-Tasin Yongngakesone, Ikenna C. Eze, Medea Imboden, Banchob Sripa, Daniel Reinharz and Nicole Probst-Hensch

Краткий обзор
Предпосылки. В результате эпидемиологического перехода, системы здравоохранения в странах с низким и средним уровнем доходов столкнулись с двойным бременем инфекционных заболеваний и возникновением неинфекционных болезней. О взаимовлиянии этих двух групп заболеваний известно мало. Целью данного исследования было изучение одновременного наличия гельминтозов и сахарного диабета у взрослых из Лаосской Народно-Демократической Республики (Лаосская НДР).
Методы. Мы провели кросс-секционное исследование среди 1600 взрослых людей старше 35 лет, выбранных случайным образом, из четырех разных социально-экономических и экологических регионов. Информация о социально-демографических факторах, факторах риска и санитарно-гигиенических условиях была получена при личном собеседовании. Также были проведены клинические обследования, которые включали сбор данных антропометрии (рост, вес, ширина талии и бедер) и данных измерения артериального давления. Случаи диабета классифицировались на основании диагноза, который сообщили сами пациенты, и анализа, выполненного у постели больного, на HbA1c из образцов, полученных при прокалывании пальца. С помощью эфир-формалинового метода был проведен анализ на наличие в кале яиц гельминтов для выявления кишечных паразитарных заболеваний. Независимая связь гельминтозов с диабетическим статусом и наличием HbA1c оценивалась с использованием множественного регрессионного анализа.
Результаты.Распространенность преддиабета и диабета составила 37,3 % и 22,8 % соответственно. Пятьдесят шесть процентов случаев диабета являлись неразспознанными, а в 85 % распознанных случаев диабета отмечали неудовлетворительный контроль гликемии. У пациентов из сельских районов и южных районов страны наблюдался более высокий уровень заболеваемости инфекцией *Opisthorchis viverrini (беличья двуустка)*, которая представляет собой наиболее распространенный гельминтоз (30,5 %). Мы обнаружили положительную связь между инфекцией, вызванной *Taenia* spp., анализом на HbA1c (β = 0,117, 95 %, *ДИ*: 0,042–0,200) и риском возникновения сахарного диабета (*OR* = 2,98; 95 %, *ДИ*: 1,10–8,05). Связи инфекций, вызванных другими видами гельминтов, с наличием HbA1c выявлено не было.
Выводы. Показатели гипергликемии и диабета в Лаосской НДР являются тревожно высокими, но согласуются с другими высокими показателями в регионе. Учитывая высокие показатели гиподиагностики и плохо контролируемую гликемию у пациентов с СД, для местной системы здравоохранения важен периодический скрининг и лечение СД. Для поиска причинно-следственных факторов риска возникновения СД в регионе необходимы многочисленные многолетние данные в когортных группах с включением биомаркеров. Маловероятно, что с помощью информации о распространенных кишечных инфекциях гельминтов, включая *O. viverrini,* можно объяснить высокие показатели СД.

Translated from English version into Russian by Alexander Vareiko and Tatiana Com, through

Asociación entre las infecciones por helmintos y la diabetes mellitus en adultos de la República Democrática Popular Lao: un estudio transversal

Nan Shwe Nwe Htun, Peter Odermatt, Phimpha Paboriboune, Somphou Sayasone[1] , Melisa Vongsakid, Phimolsarn-Nusith Vilayouth, Xuan Duong Tran, Phoum-Savath Younnavong, Navalone Andriama-Hefasoa, Nilun-Done Senvanpan, Anousine Homsana, Baocher Lianosay, Dalouny Xayavong, Dimbitsoa Rakotomalala Robinson, Phaivanh Bounsavath, Phoy-Phaylinh Prasayasith, Seng-Davanh Syphan, Yi-Xiao Lu, Kanchana Thilakoun[3], Xaipa-Song Xaiyaphet, Phout-Tasin Yongngakesone[3], Ikenna C. Eze, Medea Imboden, Banchob Sripa, Daniel Reinharz and Nicole Probst-Hensch

Resumen

Contexto: Debido a la transición epidemiológica, los sistemas de salud en los países de ingresos bajos y medios se enfrentan crecientemente a la doble carga de enfermedades infecciosas y de enfermedades emergentes no transmisibles. Se sabe muy poco sobre la interacción entre estos dos grupos de enfermedades. Este estudio pretendía investigar la coocurrencia de las infecciones por helmintos y la diabetes mellitus en adultos de la República Democrática Popular Lao (Laos).

Métodos: Hemos llevado a cabo un estudio transversal con 1.600 adultos de 35 años o mayores, procedentes de cuatro provincias con diferentes condiciones socioeconómicas y ecológicas. La información sobre los perfiles sociodemográficos, factores de riesgo y condiciones de salud se obtuvo a través de entrevistas personales. También se realizaron ensayos clínicos, entre ellos antropometría (altura, peso, circunferencia de cintura y cadera) y mediciones de la presión arterial. La clasificación de la diabetes se basó en autodiagnósticos y pruebas de HbA1c en el punto de atención mediante punciones digitales. Las muestras fecales para el diagnóstico de helmintos se examinaron mediante el método de concentración de formol-éter, una técnica utilizada en el diagnóstico de infecciones intestinales parasitarias. Las asociaciones independientes entre infecciones por helmintos y casos de diabetes y HbA1c se evaluaron mediante múltiples análisis regresivos.

Resultados: La prevalencia de prediabetes y diabetes fue del 37,3% y 22,8%, respectivamente. Un cincuenta y seis por ciento de los casos de diabetes no fueron diagnosticados y un 85% de los diagnosticados mostraron un control glucémico deficiente. Los participantes provenientes de áreas rurales y del sur del país presentaron índices de infección más altos, siendo *opisthorchis viverrini* el causante de infección por helmintos más común (30,5%). Encontramos una asociación positiva entre infecciones por *Taenia* spp. y HbA1c ($\beta = 0{,}117$; 95% *IC*: 0,042–0,200) y riesgo de DM (*OR* = 2,98; 95% *IC*: 1,10–8,05). Ninguna otra especie helmíntica se vinculó con HbA1c.

Conclusiones: Los índices de diabetes e hiperglucemia en Laos son alarmantemente altos, pero concuerdan con otros índices elevados de la región. Dado el gran número de infradiagnósticos y de glucemia con control deficiente en pacientes con DM, es esencial que los sistemas de salud locales lleven a cabo pruebas y tratamientos de DM de forma rutinaria. Se necesitan estudios exhaustivos de cohortes longitudinales que incorporen biomarcadores en la búsqueda de factores de riesgo causales de DM en la región. Es improbable que las infecciones por helmintos intestinales comunes, como *O. viverrini*, expliquen los elevados índices de DM observados.

Translated from English version into Spanish by Aurora Sevilla and Macarena Belén Pierrot, through
