# Supplementary material for: Association between helminth infections and diabetes mellitus in adults from the Lao People’s Democratic Republic: a cross-sectional study
Source: Infect Dis Poverty. 2018 Nov 6;7:105. doi: 10.1186/s40249-018-0488-2 (PMC6219195; doi:10.1186/s40249-018-0488-2)
Supplement: Supplementary file 2 — Independent association of single infections and infection groups with HbA1c in participants with HbA1c levels in the normal range (columns to the left) (N = 609) and in the normal and prediabetic range (columns to the right) (N = 1179), excluding participants self-reporting a physician-diagnosis of DM. (PDF 80 kb) [file 40249_2018_488_MOESM2_ESM.pdf]

Additional file 1: Independent association of single infections and infection groups with HbA1c in participants with HbA1c levels in the normal range (columns to the left) (N=609) and in the normal and prediabetic range (columns to the right) (N=1,179), excluding participants self-reporting a physician-diagnosis of DM

**Infections**	**Adjusted for other infections, age, gender, study sites, SES status, education status, smoking status, alcohol consumption and hemoglobin level**		**Additionally adjusted for BMI and physical inactivity**		**Adjusted for other infections, age, gender, study sites, SES status, education status, smoking status, alcohol consumption and hemoglobin level**		**Additionally adjusted for BMI and physical in activity**	
	**β**	**95% CI**	**β**	**95% CI**	**β**	**95% CI**	**β**	**95% CI**
**Grouped infection**[1]								
**Nematodes**	0.009	-0.003 0.022	0.008	-0.004 0.021	0.009	-0.005 0.022	0.010	-0.003 0.023
**Trematode infection**	0.004	-0.004 0.011	0.003	-0.004 0.010	-0.003	-0.011 0.004	-0.003	-0.010 0.005
**Cestodes** *(Taenia* sp.)	**-0.049**	**-0.075 -0.022**	**-0.049**	**-0.075 -0.022**	-0.022	-0.050 0.006	-0.020	-0.046 0.007
**Single infections**[2, 3]								
Opisthorchis viverrini	0.002	-0.005 0.100	0.002	-0.005 0.009	-0.005	-0.014 0.003	-0.005	-0.013 0.004
Hookworm	0.009	-0.006 0.024	0.009	-0.006 0.023	0.0006	-0.017 0.018	0.0009	-0.016 0.018
Minute intestinal flukes	0.006	-0.010 0.021	0.004	-0.010 0.020	0.012	-0.003 0.028	0.012	-0.003 0.028
Strongyloides stercoralis	-0.002	-0.025 0.023	0.0004	-0.023 0.024	0.009	-0.013 0.031	0.015	-0.007 0.036
Trichuris trichiura	0.012	-0.030 0.054	0.007	-0.035 0.049	0.021	-0.027 0.069	0.017	-0.030 0.063
Taenia sp.	**-0.049**	**-0.075 -0.022**	**-0.049**	**-0.075 -0.023**	-0.022	-0.050 0.006	-0.020	-.046 0.007

[1]Model including infection groups nematodes (yes vs. no), trematode infection (yes vs. no), and cestodes (yes vs. no)

[2]Model including single infections hookworm (yes vs. no), *O. viverrini* (yes vs. no), minute intestinal flukes (yes vs. no) and cestodes (yes vs. no); subjects with other rare types of infections were excluded from this analysis.

[3]subjects with other rare types of infections were excluded from this analysis
