# Supplementary material for: Association between helminth infections and diabetes mellitus in adults from the Lao People’s Democratic Republic: a cross-sectional study
Source: Infect Dis Poverty. 2018 Nov 6;7:105. doi: 10.1186/s40249-018-0488-2 (PMC6219195; doi:10.1186/s40249-018-0488-2)
Supplement: Supplementary file 3 — Independent association of single infections with pre-DM and DM status combined and compared to non-DM, with and without adjustment for BMI and physical inactivity, excluding participants self-reporting a physician diagnosis of DM and intake of DM medication (N = 14661) (PDF 80 kb) [file 40249_2018_488_MOESM3_ESM.pdf]

Additional file 2:Independent association of single infections with pre-DM and DM status combined and compared to non-DM, with and without adjustment for BMI and physical inactivity, excluding participants self-reporting a physician diagnosis of DM and intake of DM medication (N=1,466[1])

**Pre DM and DM status vs. Normal[2]**	**OR[3]**	**95% CI**	**p-value**	**OR[4]**	**95% CI**	**p-value**
Opisthorchis viverrini	0.77	0.59-1.00	0.27	0.82	0.62-1.07	0.14
Hookworm	0.74	0.44-1.26	0.53	0.78	0.46-1.33	0.37
Minute intestinal flukes	1.50	0.92-2.44	0.10	1.47	0.89- 2.43	0.13
Strongyloides stercoralis	1.39	0.66-2.92	0.38	1.73	0.80-3.74	0.16
Trichuris trichiura	0.77	0.16-3.73	0.75	0.67	0.13-3.52	0.64
Taenia sp.	1.56	0.70-3.46	0.28	1.65	0.70-3.46	0.23
Age	1.05	1.04-1.06	**<0.001**	1.05	1.04-1.06	**<0.001**
Gender (Male)	1.42	1.04-1.94	**0.03**	1.27	0.92-1.75	0.14
Study sites(Vientiane as reference)						
Lung Prabang	0.74	0.53-1.03	0.07	0.77	0.55-1.10	0.14
Saravane	0.74	0.52-1.06	0.10	0.84	0.58-1.22	0.35
Champasack	0.71	0.51-0.99	**0.04**	0.79	0.57-1.11	0.18
Socio-economic status(Poorest as reference)						
Second least poorest	1.16	0.88-1.53	0.30	1.03	0.77-1.37	0.30
Least poorest	1.35	1.00-1.82	**0.04**	1.14	0.84-1.56	0.40
Ever smokers	1.17	0.87-1.57	0.31	1.32	0.97-1.79	0.08
Education(Illiterate)						
Primary	0.92	0.57-1.47	0.72	0.81	0.57-1.16	0.25
Secondary	0.85	0.49-1.45	0.55	0.82	0.55-1.22	0.33
Ever Alcohol drinkers	0.62	0.49-0.81	**<0.001**	0.59	0.46-0.77	**<0.001**
Hb concentration	1.02	1.01-1.02	**<0.001**	1.01	1.00-1.02	**0.005**
+ BMI	-	-	-	0.98	0.72-1.33	0.90
+ physical activity	-	-	**-**	0.00	0.00-0.01	**<0.001**

[1] subjects with other rare types of infections were excluded from this analysis
[2] categorization on a positive self-report of DM diagnosis and otherwise, based on HbA1c concentrations
[3] mutually adjusted for variables listed
[4] additionally adjusted for BMI and physical inactivity
